# netANOVA: novel graph clustering technique with significance assessment via hierarchical ANOVA

**DOI:** 10.1093/bib/bbad029

**Published:** 2023-02-04

**Authors:** Diane Duroux, Kristel Van Steen

**Affiliations:** BIO3 - Systems Genetics, GIGA-R Medical Genomics, University of Liege, Liege, Belgium; BIO3 - Systems Genetics, GIGA-R Medical Genomics, University of Liege, Liege, Belgium; BIO3 - Systems Medicine, Department of Human Genetics, KU Leuven, Leuven, Belgium

**Keywords:** Network clustering, System medicine, Stratified medicine, Graph comparison

## Abstract

Many problems in life sciences can be brought back to a comparison of graphs. Even though a multitude of such techniques exist, often, these assume prior knowledge about the partitioning or the number of clusters and fail to provide statistical significance of observed between-network heterogeneity. Addressing these issues, we developed an unsupervised workflow to identify groups of graphs from reliable network-based statistics. In particular, we first compute the similarity between networks via appropriate distance measures between graphs and use them in an unsupervised hierarchical algorithm to identify classes of similar networks. Then, to determine the optimal number of clusters, we recursively test for distances between two groups of networks. The test itself finds its inspiration in distance-wise ANOVA algorithms. Finally, we assess significance via the permutation of between-object distance matrices. Notably, the approach, which we will call netANOVA, is flexible since users can choose multiple options to adapt to specific contexts and network types. We demonstrate the benefits and pitfalls of our approach via extensive simulations and an application to two real-life datasets. NetANOVA achieved high performance in many simulation scenarios while controlling type I error. On non-synthetic data, comparison against state-of-the-art methods showed that netANOVA is often among the top performers. There are many application fields, including precision medicine, for which identifying disease subtypes via individual-level biological networks improves prevention programs, diagnosis and disease monitoring.

## Introduction

Subjects or objects can often be linked to systems, and studying the differences between their corresponding system representations is of particular interest to precision medicine. Examples of systems in biology include the nervous system, the circulatory system and the respiratory system. Graphs lend themselves perfectly to visualize systems [1]. A graph consists of nodes and edges as primary building blocks. Only the characteristics of these elements may differ since they can be labelled, attributed, weighted and directed (see Section 2.1). Sometimes, the term ‘graph’ may be reserved to describe an abstract data structure, whereas the term ‘network’ would refer to a concretization of a graph. Here, the terms graph and network are interchangeably used. Graph-based machine learning [2] has already been used to disentangle complex diseases and improve personalized care. Lung cancer was predicted from a protein–protein interaction (PPI) network integrated with gene expression data using a combination of spectral clustering and deep learning methods [3]. Breast cancer subtype classification was performed from PPI networks enriched with gene expression data via the integration of deep learning methods and a relational network [4].

Most of the graph analyses for complex diseases aggregate information across a whole cohort, failing to detect individual characteristics [5]. Exploiting individual-specific interactions rather than population-level systems will capture the heterogeneity between individuals and enhance the identification of new biomarkers for precision medicine. This observation paves the way for developing individual networks, where nodes and/or edges are individual-specific. For each individual, nodes are variables (e.g. genes), and edges show the link between these variables for that individual.

In this work, we want to understand what makes (individual) networks different. We aim at comparing entire networks to create groups of graphs that are homogeneous. In other words, we start from a set of graphs, and we are interested in finding sub-groups of graphs to learn about their different characteristics and examine the driving factors for similarity or dissimilarity. Unsupervised learning is required as in the medical context, grouping labels are not necessarily known and the goal is to discover discriminating properties among the data. The number of classes may not be known either, so the algorithm has to derive it. Finally, the method needs to include notions of statistics to assess that the groups are significantly different.

The advancement of machine learning has led to the emergence of various network analytics tools and techniques [2, 6]. In this work, we focus on the scenario where we have a list of graphs as input and aim to create groups of similar graphs. One option is to represent the edge weights as a vector and use these vectors as input to downstream analyses. This approach is easy to implement but ignores the topology of networks and is restricted to situations where networks have the same set of nodes. Another possibility is to derive graph summary statistics (e.g. average degree and path length). This method has proven successful ([7, 8]) but tends to ignore local structures. To take into account local dissimilarities, an alternative is to apply network-specific distances [9] or graph kernels [10] to estimate the similarity/dissimilarity between networks and use the network similarities in kernel-based ML methods to identify groups of homogeneous graphs. However, the number of groups may not be known a priori, so there is a need to incorporate an algorithm that derives it. Also, this method often suffers from a high computational burden. Deep learning methods can help solve this scalability issue while bringing strong performance. Initially, these methods were constructed to work on vectors. Graph neural networks (GNNs) [11] extend them to graphs. GNNs include graph embedding and graph convolutional networks (GCNs).

Graph embedding aims at computing a fixed-size vector representation of a graph to decrease dimensionality. Structural properties in the embedding should correspond to the properties of the networks. For instance, InfoGraph [12] maximizes the mutual information between the graph-level representation and the representations of substructures of different scales (e.g. nodes, edges, triangles). In the GraPHmax approach [13], the concept of periphery representation of a graph into a single framework is introduced and combined with hierarchical GNNs and mutual information maximization. The graph2vec algorithm [14] extends document embedding neural networks by considering an entire graph as a document and the rooted subgraphs (i.e. non-linear substructures) around every node in the graph as words, to create embeddings of entire graphs. With all these approaches, the derived representations in the embedding space can be used for classification (e.g. elastic net, SVM-L1, signal subgraph, dlda, lasso) or clustering (e.g. hierarchical clustering, $k$-means, spectral clustering). Notably, classification has received a lot of attention. However, in many fields, group labels are not known, and unsupervised learning is required. Also, deriving the optimal number of clusters is often decoupled from the mainstream analysis [13].

GCNs adapt convolutional neural network methodologies for graph-structured data. To provide a network representation similar to the image convolution, GCN algorithms use a spectral [15], or spatial-based [16] convolution over the graph. GNNs’ drawbacks include their lack of interpretability which is an important issue for instance in biology where the goal is to understand the processes involved in the system studied [17], and in precision medicine, where physicians will need to understand the prediction to trust it [18]. However, some progress has been made recently [19]. Furthermore, GNNs require a large amount of data to provide accurate predictions. It can be an issue in personalized medicine where it is complex to collect large samples for feasibility and privacy reasons [20]. Deep learning methods for graph clustering have been shown to achieve high performance [21–23]. As clustering methods are typically driven by particular characteristics of the data, no holy grail generic method is likely to prevail. Wu *et al*. [24] showed that improved performance can be obtained with more traditional graph clustering approaches over deep learning ones in specific scenarios (in their work, the WL-CT kernel).

In response to the illustrated shortcomings, with our novel netANOVA analysis workflow we aim to exploit information about structural and dynamical properties of networks to identify significantly different groups of similar networks. We do so by developing a novel group comparison testing workflow that sequentially evolves down a hierarchical tree. The netANOVA test statistic relies on additive partitioning rather than centroids; the latter is typical in traditional analysis of variance (ANOVA) hypothesis testing [25]. Statistical significance is assessed empirically to avoid reliance on distributional assumptions. Furthermore, our flexible analysis workflow accommodates small datasets (smaller than $20$) as well as larger ones (up to a few thousand), and can be used in multiple contexts via customizable hyperparameter settings, handling weighted, sparse or multi-layered networks.

In summary, our analysis workflow can be used to identify and formally test for differences between objects that can be represented as graphs. Hence, application areas include, but are not restricted to, precision medicine and the challenging task of identifying endotypes for biomarker development.

## Materials and methods

### Network and graphs

A network is a data structure consisting of nodes and edges modelling the relations between two nodes. A network $G$ can be defined as $G=(V, E)$, where $V$ is the set of nodes, and $E$ are the edges between them. In biology, nodes can be genes, messenger RNAs, proteins or metabolites, and edges can represent molecular regulation, genetic interactions, co-localization or co-occurrence.

For binary networks, a graph is completely described by its adjacency matrix $A\in{0,1}_{n \times n}$, where $A(i,j) = 1$ if and only if the link $(i,j)\in E$. If matrix $A$ is symmetric, then the graph is undirected, otherwise directed. For weighted networks, $A(i,j)=w_{ij}$, with $i,j \in N$. Attributed networks have labels and/or attributes on the nodes and/or edges. Attributes (resp. labels) are commonly expected to be real values (resp. alphabetic values).

### Distances and similarities between networks

Distance and similarity are related concepts: when distance increases, similarity decreases. A ‘distance metric’ is a function that satisfies the non-negativity, identity, symmetry and triangle inequality properties [26]. Often, some properties are not necessary, and a ‘distance measure’ may be used. The latter also captures how different two objects are but is a function that does not satisfy at least one of the four properties. A similarity function satisfies the non-negativity, boundedness, identity and symmetry properties. A distance can be calculated based on similarity and vice versa. NetANOVA is based on a distance matrix (Algorithm 2). Hence, when the link between networks is directly computed using a distance measure (e.g. the edge difference distance or the hamming distance), no additional transformation is needed. However, when similarities are used to study the link between networks (e.g. with the shortest path kernel or the random walk kernel), we need to convert them into distances. Specifically, when a similarity is computed via a kernel, then the distance between two networks $G_1$ and $G_2$ can be calculated as the difference between the self-similarities $K(G_1, G_1) + K(G_2, G_2)$ and the cross-similarity $K(G_1, G_2)$ [27]: $d(G_1, G_2) = K(G_1, G_1) + K(G_2, G_2) - 2K(G_1, G_2)$. The multiplicative factor 2 is needed to ensure that d(G, G) = 0.

The choice of distance and similarity measures is a critical step in clustering efforts. An extensive range of graph comparison measures exists. Requiring time-computational efficiency when clustering a large number of graphs dramatically reduces the options. Moreover, most of the remaining distances handle undirected [28–30] and unweighted [31–33] networks only. Hence, defining a distance between graphs is a cumbersome task, which requires seeking a context-dependent balance between computational efficiency, performance and interpretability. Following Tantardini *et al*. [9], we group network-based distances into two main classes: Known Node-Correspondence (KNC) and Unknown Node-Correspondence (UNC) methods.

In the KNC scenario, the networks have the same set of nodes or at least a common subset, and the pairwise correspondence between the networks nodes is known. In other words, a distance requires node correspondence when some meaningful mapping between the node sets of the graphs exists. Typically, there is Known Node-Correspondence when networks come from the same application field. KNC distances gather all the methods, such as Euclidean, Jaccard or DeltaCon distances, which require a priori to know the correspondence between the nodes of the compared networks. These methods allow comparing networks where nodes are labelled and hence not exchangeable.

UNC approaches do not require knowledge of the correspondence between nodes. UNC methods, such as spectral distances, graphlet-based measures and Portrait Divergence, are suited for global structural comparison. They indicate how much the structures of graphs differ. We will pay special attention to graph kernel measures [10]. A kernel is a measure of similarity between objects and must satisfy two mathematical requirements: it must be symmetric and positive semi-definite. Notably, there are much more UNC approaches than KNC ones.

Our netANOVA workflow accommodates multiple measures: the edge difference distance [34], a customized KNC version of $k$-step random walk kernel (see Supplementary) [35], DeltaCon [36], GTOM [37] and the Gaussian kernel on the vectorized networks [38] are proposed as KNC methods. The Hamming distance [39], Shortest path kernel [40], $k$-step random walk kernel and Graph Diffusion Distance [34] are optional UNC methods. More details about these distance and similarity measures, and the reasoning behind these choices are given in Supplementary.

### Identification of homogeneous subgroups

Distance-based clustering evolves around finding homogeneous subgroups of objects, where objects with minimal distances between them are assigned to the same cluster. The two most popular distance-based clustering approaches are hierarchical clustering and $k$-means clustering. The first clusters objects sequentially, via inter-cluster distances. The latter classifies objects into subgroups via inter-cluster variances that need to be minimized. Hierarchical clustering has the additional advantage that a tree (dendrogram) visualizes different granularities in the clustering process, which we will exploit in our workflow.

NetANOVA is built around hierarchical distance-based clustering, with distance measures as in Section 2.2. We use the standard agglomerative clustering which first considers each object as a cluster and then merges clusters successively until one cluster contains all objects.

### Deriving the optimal number of clusters

**Figure 1:**
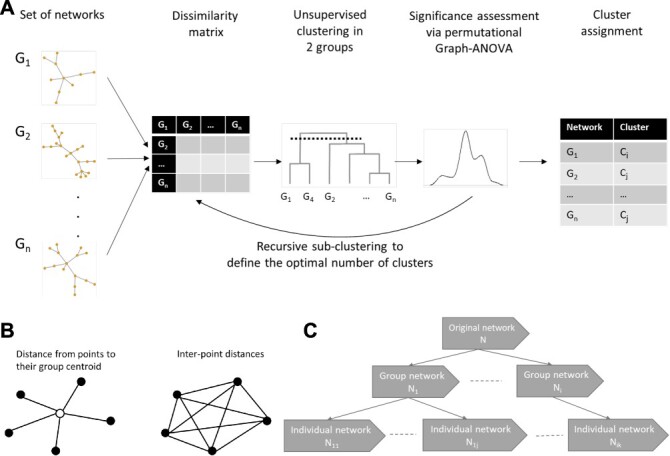
**(A)** NetANOVA workflow. Starting from the list of networks, the pairwise distance between each pair of networks is computed. Then a hierarchical clustering is applied to the distance matrix to derive a dendrogram and identify preliminary groups. Algorithm 2 is applied to the two first groups (from the top to the bottom of the dendrogram). If the two first groups are statistically different, Algorithm 2 is applied to each of the two subgroups. Recursively (Algorithm 1), a tree is built to derive the optimal number of clusters, with two stop conditions: the groups are too small, or the groups are not significantly different. **(B)** The sum of squared distances from individual points to their centroid is equal to the sum of squared interpoint distances divided by the number of points. **(C)** Simulation set-up. An original network with $m$ nodes and density $d$ is generated. The baseline network has a random structure (simulated from the Erdos–Renyi model), a density of 0.05, 100 nodes and binary edges. Group networks are derived by perturbing (rewiring, adding or removing edges) the original graph. Individual networks are derived by perturbing the group networks.

To determine the optimal number of clusters, we recursively test for distances between two groups of networks, progressing from the root node to the end nodes of the clustering dendrogram (Figure 1**(A)**). Many clustering methods require the user to pre-specify the number of clusters. However, this information is often not known. Incorrect estimation will prevent learning the real clustering structure. Here, the algorithm derives the number of classes. If the two groups created from a node of the dendrogram are statistically different, the algorithm to find the optimal number of clusters proceeds in the child nodes (Algorithm 1). Details about the underlying formal hypothesis test are given next (Section 2.5). There are two stopping conditions: the two subgroups are too small or are not statistically significantly different. The first requires setting a threshold for the minimum allowable size of a subgroup. The result is a decision tree where the end leaves are the final clusters, and splitting is based on a formal group comparison test between network collections. Note that when one of the two groups tested ($a$ and $b$) has a size not surpassing the minimum size threshold (for example group $a$), the statistical test is applied to the other group (group b giving rise to subgroups $b_1$ and $b_2$). If subgroups $b_1$ and $b_2$ are statistically different, group $a$ is regarded to be outlying and hence an independent group.


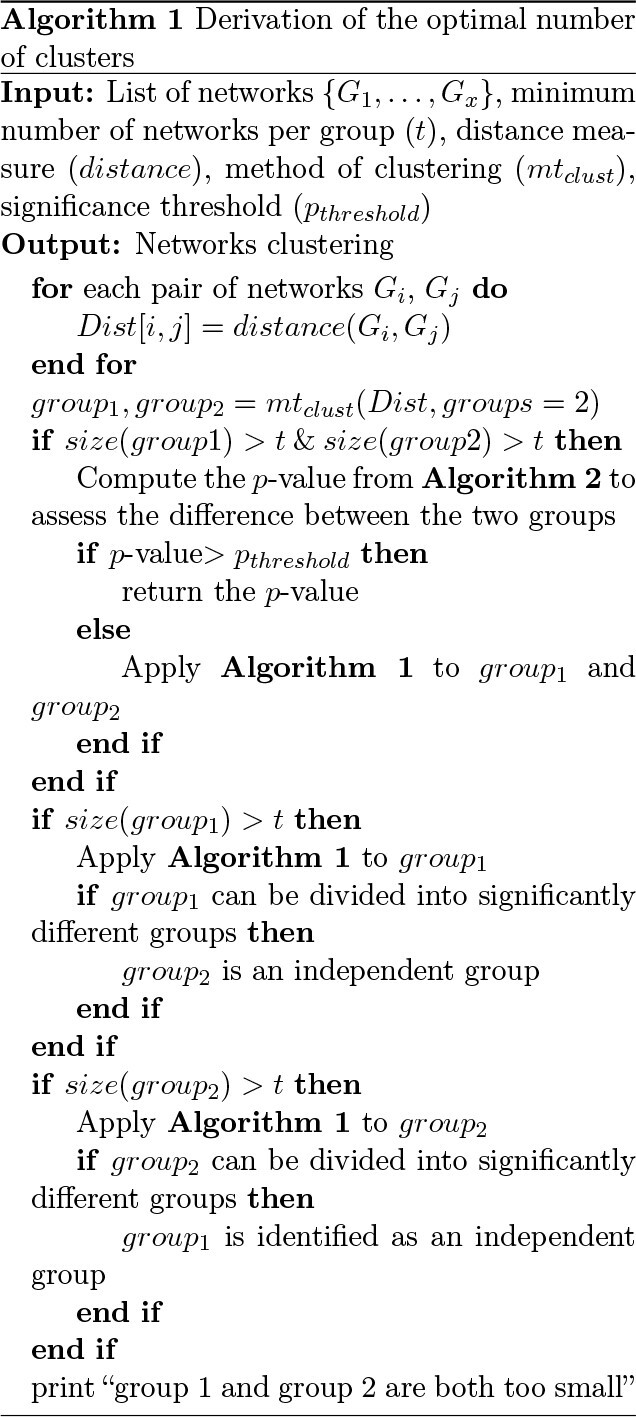


With the aforementioned sequential procedure, false-positive control is a concern. We include two options to correct for multiple testing. First, we correct the *P*-values using the depth of the tree, i.e. no correction at the root node, $p_{adj}=p\times 2$ at level 2 of the dendrogram, $p_{adj}=p\times 3$ at level 3 of the dendrogram, and so forth. If at a node of the dendrogram, the difference between the two associated groups is not tested because one of the two groups is smaller than the minimum group size threshold, then the level (i.e. depth of the dendrogram) is not incremented. Also, we implement the correction developed by Meinshausen [41] and created for variable selection. It controls the FWER at level $\alpha \in (0, 1)$, by performing the hypothesis test described in Section 2.5 at each node $j$, with the significance threshold $\alpha _{adj}=\alpha \times \frac{N_j-1}{N-1}$ with $N_j$ the number of networks clustered at node $j$ and $N$ the total number of networks. It gives increased power to the first nodes (near the root) of the tree. Also, we include the possibility of not correcting for multiple testing in the workflow. Strikingly, computing the total number of tests and applying a Bonferroni correction to each test to keep FWER under control would bypass the hierarchical structure of the analysis.

In Section 3.1, we evaluate these multiple testing corrections for FWER control. We define FWER of the entire workflow as the probability of falsely rejecting the null hypothesis at least once when moving down the fixed hierarchical tree.

### A novel network-based empirical testing strategy

The netANOVA compares the variation within a group of graphs and the variation between groups of graphs, using the ratio of the F-statistic [42]. The higher the value of F, the more likely the null hypothesis $H0$ of no difference among the group means is false. In univariate ANOVA, the total sum of squares (SST) is computed from sums of squared differences between observations and their group mean (SSW), and between group means and the overall sample mean (SSA). A multivariate ANOVA is derived by adding up the sums of squares across all variables. Hence, a classical ANOVA test uses the concept of the mean of a group, which is complex for networks. To overcome this issue, we take advantage of the following property [25]: the sum of squared distances between points and their centroid is equal to the sum of squared interpoint distances divided by the number of points (Figure 1**(B)**).

Therefore, the total sum of square can be expressed as

(1) \begin{align*}& SST=\frac{1}{N}\sum_{i=1}^{N-1}\sum_{j=i+1}^{N}d^2_{G_i,G_j}. \end{align*}

The within-group sum of squares is

(2) \begin{align*}& SSW=\sum_{l=1}^{k}\sum_{i=1}^{N-1}\sum_{j=i+1}^{N}\frac{1}{n_l}e_{ijl}\times d^2_{G_i,G_j}. \end{align*}

The among-group sum of squares is

(3) \begin{align*}& SSA=SST-SSW. \end{align*}

Finally, the F-ratio is

(4) \begin{align*}& F=\frac{SSA/(k-1)}{SSW/(N-k)}, \end{align*}

with $N$ the total number of individuals, $n_l$ the number of networks in group $l$, $k$ the number of groups, $d_{G_i,G_j}$ the distance between graph $i$ and graph $j$, and $e_{ijl}$ takes the value 1 if network $i$ and network $j$ are in the group $l$, and 0 otherwise.

Another benefit of not using a mean relates to the distance used. For Euclidean distances, the mean for each variable across observations within a group constitutes a measure of central location of the group. This is not true for many non-Euclidean distances. The statistic is also interesting in terms of the computational burden. Even though the distance between each pair of networks is required, it is computed only once. No additional computation is required on permutation replicates. In contrast, traditional ANOVA settings would require repetitive computation of network averages and distances to network averages.

Since the actual statistic distribution may not have a closed form and distributional assumptions may not hold on large samples, significance is derived via permutation replicates. One critical assumption for this test is that the observations need to be exchangeable under a true null hypothesis. Thus, one needs to be careful regarding the interpretation of the significance assessment to ensure that the difference between groups is not due to differences in dispersion (i.e. difference in the distributions). Permutation tests in standard ANOVA settings typically rely on permuting known group labels. In our context, group labels are a priori unknown and inferred via a clustering procedure. Group label reshuffling, conditioning on two clusters in a clustering, will inflate overall type I error [43]. To circumvent this we apply the following procedure to create appropriate null distributions of test statistics. Instead of permuting group labels at each dendrogram node, we permute the distances between the investigated graphs and re-apply hierarchical clustering to identify two groups. If both groups have a size surpassing the group size threshold, we compute the statistics described above. For instance, we repeat the procedure 99 times and compare the permuted statistics $F^\pi $ with the observed statistic $F$:

(5) \begin{align*}& p-value=\frac{\# (F^\pi \geq F)+1}{Total \# F^\pi + 1} \end{align*}

We emphasize that when permuting the values in the original distance matrix, the new matrix cannot be considered a distance matrix because the measure violates the triangular inequality. After applying the permutations, we can indeed obtain $d_{G_i,G_l}>d_{G_i,G_k} + d_{G_k,G_l}$. The linkage criteria in the hierarchical clustering are then limited to methods requiring dissimilarities to be non-negative and symmetric only, such as complete and average linkage methods [44]. The evaluation of the impact of this linkage criteria is shown in Section 3.1. In the available code, the user can select ‘complete’ (default) or ‘average’ linkage.

In Section 3.1, we also compare different perturbation levels of the distance matrix and set the default amount of perturbation in the distance matrix to 20% and the default number of replicates to 99. These parameters are customizable, as is the significance threshold (default 0.05).

### Evaluation and application

All the experiments are conducted on a Scientific Linux release 7.2 (Nitrogen) cluster.

#### Simulations—Type I error

To evaluate the statistical relevance of the detected groups and the impact of our significance assessment, we study if the proposed workflow controls the Type I error. We perform a simulation analysis based on 1000 replicates for that purpose. First, we generate an original random graph with $m$ nodes and a density $d$. For weighted networks, we simulate binary networks and replace the value of edges present by a random number from a normal distribution with a mean of 0.5 and standard distribution of 0.5*0.5. The edge values are scaled via the min–max scaling algorithm so that values of the adjacency matrix range from 0 and 1. Importantly, we consider the minimum and maximum values across all objects, so these boundaries are the for all networks.

Then, in both the binary and the weighted contexts, we derive $n$ graphs by randomly rewiring the edges while preserving the original graph’s degree distribution [45] of the original graph. Specifically, the algorithm chooses two arbitrary edges (($N_a$,$N_b$) and ($N_c$,$N_d$)) and substitutes them with ($N_a$,$N_d$) and ($N_c$,$N_b$) if they do not yet exist.

We evaluate the impact on the type I error of the level of perturbation, the number of graphs, the number of nodes, the graph density, the minimum group size, the distance used to compute dissimilarity between graphs and the minimum number of networks per group. In the baseline, the original network has a random structure (simulated from the Erdos–Renyi model), generated using the function erdos.renyi.game() from the R package igraph [45]. It has a density of 0.05, 100 nodes and binary edges. When at least two groups are detected via netANOVA in a permutation, that permutation is considered a false positive (FP). This allows us to compute the type I error rate as $\frac{ \text{\# FP}}{1000}$.

#### Simulations—power

We simulate the situation where each network represents its own individual (e.g. patients), the nodes are labelled and shared across all networks (e.g. genes) and several populations exist (e.g. disease sub-type). The goal is to identify and compare the different populations. To this end, the following experimental set-up (Figure 1**(C)**) is implemented. First, we generate an original network and perturb it to derive group networks. Then, we perturb each group network to create individual networks. The goal is to apply the unsupervised netANOVA to assign the individual networks to the correct groups. We validate the clustering via Jaccard similarity.

We consider the same baseline original network as in Section 2.6.1: a network with a random structure, a density of 0.05, 100 nodes and binary edges. In the baseline, we switch 40% of the original edges while preserving the degree distribution. This is done 10 times to create 10 group networks. Then, we switch 40% of the edges for each group network while preserving the degree distribution, 10 times to create 10 individual networks per group.

We create 800 replicates, and we evaluate the impact of multiple parameters. Some parameters are associated with the network properties, such as network size or structure. Others are related to the method, such as the correction for multiple testing or the distance between graphs. The influence of the perturbation types and the minimum size of the groups are also studied.

#### Real-life data application

We apply netANOVA to two real-life bioinformatics graph datasets. For both applications, we use datasets with known clusterings to be able to use these ‘true’ clusters to compute the performance of netANOVA. Hence, a supervised model could be used to define group membership. Still, the goal here is to evaluate the unsupervised procedure; we do not use any information about the groups in the netANOVA workflow.

#### UNC scenario

The graph dataset MUTAG [46] contains collection of nitroaromatic compounds. The aim is usually to predict the mutagenicity of the compounds on Salmonella typhimurium. The nodes represent atoms, while edges are bonds between the corresponding atoms. The dataset includes 188 samples of chemical compounds. It is publicly available and commonly used to compare classification performances.

#### KNC scenario

We also apply netANOVA to graphs with known node correspondence, i.e. multiplex networks. Previous work [47, 48] has shown the potential of brain networks to distinguish between various brain disorders. We selected the COBRE brain networks [47, 49]. It contains 124 individual-specific networks: 70 controls and 54 schizophrenics. The brain networks are constructed from imaging data (resting state fMRI) to represent functional connectivity between regions of the brain. The graphs are composed of 263 nodes obtained from the Power parcellation [50] and 34 453 edges. The edge weights are the Fisher-transformed correlation between the fMRI time series of the nodes. Nuisance covariates like age, gender, motion and handedness have been regressed out. For a description of the preprocessing steps to obtain the network edge weights, see [47].

The brain networks are fully connected. In their functional brain connectivity analysis (identification of controls versus autism spectrum disorder), Wills *et al*. [51] found that only a subset of edges represents the structural differences between the two groups of graphs studied. The dissimilarities could not be identified with all the edges. Also, since the local changes in connectivity were of the same order of magnitude as the random local variations, a comparison using all the edges was ineffective. Similar findings were reported [52, 53]. Therefore, we evaluated the impact of graphs sparsification using the method developed by Relión *et al*. [47] to select edges. This method incorporates the network nature of the data via penalties to promote sparsity in the number of nodes, in addition to sparsity penalties that encourage the selection of edges. Specifically, to capture structural predictive edges, the authors focus on convex structured sparsity penalties that favour a small number of active nodes (nodes attached to at least one edge with a non-zero coefficient). To find a set of such nodes, they focus on convex formulations that encourage small active node sets indirectly. They penalize the number of active nodes by treating all edges connected to one node as a group. Then, eliminating this group is equivalent to de-activating a node.

## Results

All adopted simulation and real-life application parameters settings and choices are summarized in Supplementary Table 1.

### Type I error

We first investigated the influence of the network properties on the type I error (see Table 1.). Some measures gave rise to a type I error under control in all experimental settings: edge difference, Hamming distance, shortest path kernel, $k$-step random walk kernel, DeltaCon distance and Gaussian kernel. The graph diffusion distance was more prone to type I error. The network density had a high impact: a higher density produces more conservative results. In our simulation setting, we first generate an original random graph, and we derive 50 graphs by randomly rewiring 40% of the original graph’s edges while preserving the original graph’s degree distribution. Hence, increasing the density will provide more information but also more heterogeneity. Indeed, including more edges may induce more noise [54]. We also evaluated the algorithm on weighted networks. Although some distances became highly conservative, most of them tended to behave as in the baseline.

**Table 1:** Type I error (%) of the netANOVA workflow depending on network properties, estimated over 1000 random replicates, as explained in Section 2.6.1. The baseline corresponds to 50 networks, each one 100 nodes, a density of 0.05 and 40% of the edges switched. The minimum group size is 10, 20% of the distance matrix is shuffled in the netANOVA permutations and the linkage method in the hierarchical clustering is ‘complete’.

Measure	Baseline	Networks	Nodes	Density	Perturbation	Weighted
		100	500	0.1	60%	
Edge difference distance [34]	4.0	4.4	1.1	1.4	4	4.7
Hamming distance [39]	4.0	4.2	1.1	1.5	4	NA
Shortest path kernel [40]	4.0	4.4	1.1	1.4	4	0
$k$ -step random walk kernel [35]	4.0	4.4	1.1	1.4	4	0
$k$ -step random walk kernel KNC [35]	2.6	4.3	1.3	1.6	4	5.2
DeltaCon [36]	2.3	3.4	1.1	1.8	3.6	3.0
Graph Diffusion Distance [34]	7.2	12.7	0.6	2.2	7.6	9.8
Gaussian kernel [38]	4.0	4.4	1.1	1.2	4.1	4.7
GTOM [37]	4.7	4.2	NA	1.4	3.9	5.1

Then, we quantified the impact of the algorithm options (see Table 2.). Overall, the type I error was still under control in almost all settings. The type I error tended to deflate when decreasing the minimum group size. Furthermore, the linkage criterium in the hierarchical clustering significantly impacted the false positive rate. The average linkage being highly conservative, the complete linkage was set as the default option. Finally, the higher the number of perturbations in the distance matrix in the netANOVA permutation procedure, the more conservative the test.

**Table 2:** Type I error (%) of the netANOVA workflow depending on netANOVA parameters, estimated over 1000 random replicates, as explained in Section 2.6.1 I error. The baseline corresponds to 50 networks, each one having 100 nodes, a density 0.05 and 40% of the edges switched. The minimum group size is 10, 20% of the distance matrix is shuffled in the netANOVA permutations and the linkage method in the hierarchical clustering is ‘complete’.

Measure	Min group size	Linkage	Perturbation of	Perturbation of
	5	average	distance matrix 10%	distance matrix 50%
Edge difference distance [34]	3.5	0	4.2	3.2
Hamming distance [39]	3.5	0.2	4.4	3.4
Shortest path kernel [40]	3.5	0	4.2	3.2
$k$ -step random walk kernel [35]	3.5	0	4.2	3.2
$k$ -step random walk kernel KNC [35]	2.1	0.1	2.5	1.6
DeltaCon [36]	2	0.2	2.2	1
Graph Diffusion Distance [34]	6.2	0.9	4.7	7.5
Gaussian kernel [38]	3.4	0	4.4	3
GTOM [37]	4.2	0.7	4.8	4

### Power

**Figure 2:**
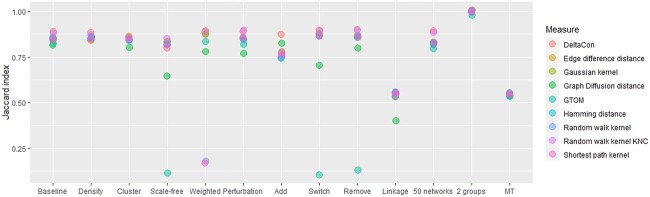
Average Jaccard index across multiple simulation scenarios for 800 replicates. The properties of networks and parameters used to derive results are described in Supplementary Table 2. The baseline scenario has an original network with a random structure, 100 nodes, a density of 0.05 and binary edges. It contains 10 groups and 10 networks per group obtained via degree preserving rewiring 40% of the edges. The hierarchical clustering is performed with complete-linkage clustering and the multiple testing correction is based on the depth of the dendrogram (Section 2.4). In the other scenarios, we altered one parameter at a time. *Density* corresponds to an original network with a density 0.1. *Cluster* corresponds to an original network with a cluster structure and *Scale-free* to a scale-free original network (from Barabasi-Albert models). *Weighted* is for weighted networks (see Section 2.6.2). *Perturbation 60%* means that group networks and individual networks are obtained with degree preserving rewiring 60% of the edges. Then, *Add*, *Remove* and *switch* correspond to addition, removal and random switching of edges instead of degree preserving rewiring. *Linkage* stands for average linkage in hierarchical clustering. The *50 networks* scenario has 10 groups of 50 networks, and the *2 groups* scenario has two groups of 10 networks. Finally, *MT* corresponds to the multiple testing correction developed by Meinshausen [41] (Section 2.4), which controls the FWER at level $\alpha \in (0, 1)$, using the significance threshold $\alpha _{adj}=\alpha \times \frac{N_j-1}{N-1}$ with $N_j$ the number of networks clustered at node $j$ and $N$ the total number of networks.

The baseline scenario has an original network with a random structure, 100 nodes, a density of 0.05 and binary edges. It contains 10 groups and 10 networks per group obtained via degree preserving rewiring 40% of the edges. The hierarchical clustering is performed with complete-linkage clustering and the multiple testing correction is based on the depth of the dendrogram (Section 2.4). The minimum group size is set to 5. In the other scenarios, we altered one parameter at a time. The properties of networks and parameters used to derive results are described in Supplementary Table 2. Overall, the baseline scenario gave good performance with a mean Jaccard index of 0.85 across all distances (see Figure 2). The correction for multiple testing using the depth of the tree (see Section 2.4) was less conservative than the correction developed by Meinshausen [41] and was, therefore, more optimal with the chosen baseline parameters. Indeed, the former detected nine groups on average across distances versus six groups for the latter. To validate the trends identified in the Section 3.1, we applied the average linkage in the hierarchical clustering. Here, it detected only seven groups on average. It confirmed that this linkage is more stringent than the complete one and makes the detection of the correct clusters more complex.

We also compared various graph characteristics. With two groups only instead of 10, the classification was perfect for almost all distances. Also, when we simulated larger groups (50 graphs per cluster), the Jaccard index was comparable with the one obtained with 10 networks per group since it ranged between 0.79 and 0.89. Then, we tested multiple perturbation types: random switching, removal of edges and addition of edges. GTOM was less indicated when the perturbation was the removal of edges (resp. random switching) since the associated Jaccard index is 0.14 (resp. 0.1) on average. We also modified the original network structure and tested scale-free graphs using Barabasi-Albert models and cluster networks. With scale-free networks, across all distances except GTOM and graph diffusion, the average Jaccard index was again relatively high (0.83). The average Jaccard index per distance ranged from 0.79 to 0.86 across all distances with cluster networks.

Moreover, we investigated the impact of perturbations within and between groups of networks by increasing this level up to 60%. The average Jaccard indexes were not highly different from those obtained with the baseline. Then, we increased the density of networks. With a density of 0.1 instead of 0.05, the average Jaccard index ranged from 0.85 to 0.88 and hence, groups were still detectable. We also tested weighted networks (see Section 3.1), and observed that distances based on random walk kernel did not perform as good as the other distances. In most settings, the graph diffusion distance tended to have difficulties clustering the graphs correctly. On the contrary, DeltaCon and the custom random walk kernel performed overall better than the other measures.

### Real-life data application

#### UNC scenario

The UNC application takes as input the list of 188 nitroaromatic compound networks. The goal is to create groups of networks to verify if we can identify the mutagenicity of the compounds on Salmonella typhimurium (2 groups). These group labels are not used to derive the clusterings, they are only used a posteriori to obtain the accuracy by comparison between the inferred groups and the ground truth. We compared the results of netANOVA with the methodologies DGI [55], InfoGraph [12], GraPHmax [13] and its variants, and graph2vec. We also applied graph2vec embedding to convert variable-size graphs into a fixed-size representation of graphs and combined it with an autoencoder. The $k$-means algorithm was used on the vector representations of the graphs obtained from the different algorithms with $k=$# unique labels$=2$. Moreover, we computed the pairwise distance between networks using the random walk kernel, and we used the inferred similarity matrix as input to a spectral clustering (with $k=2$) algorithm. We have included details on graph2vec and autoencoder parameters in the Supplementary.

**Figure 3:**
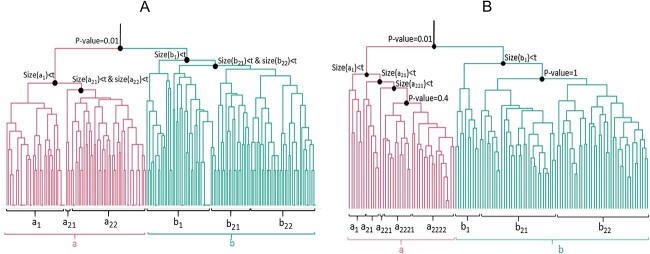
**(A)** netANOVA clustering on the MUTAG dataset. The pairwise distance between the 188 nitroaromatic compound networks is based on the random walk kernel. The minimum group size is $t=40$. We set the other parameters to the default options. Two groups are identified by netANOVA: the red and the blue groups (*P*-value$=0.01$). **(B)** netANOVA clustering on the pre-filtered COBRE dataset. The edge selection was performed according to Relión *et al*. [47], with $\rho =1$. The pairwise edge difference distance between the 124 individual-specific brain networks is computed. The minimum group size is $t=10$. We set the other parameters to the default options. Two groups are identified by netANOVA: the red and the blue groups (*P*-value$=0.01$).

The default options are selected in netANOVA. We compute the pairwise distance between the 188 nitroaromatic compound networks based on the random walk kernel. We set the minimum group size to $t=40$. Then, hierarchical clustering is applied to the distance matrix to derive a dendrogram and identify preliminary groups. We illustrate the procedure in Figure 3**(A)**. The first two groups are significantly different (Algorithm 2), so we go to the next level of the dendrogram and re-apply Algorithm 2 to assess whether the corresponding subgroups are significantly different. We progress down the dendrogram tree, and stop when the groups are too small, or when the groups are not significantly different. Since subgroup $a_1$ (Figure 3**(A)**) contains fewer networks than the minimum group size, we progress in the associated branch. Since the size of groups $a_{21}$ and $a_{22}$ are both smaller than $t$, no significant subgroup is detected. We observe the same in group $b$: Since subgroup $b_1$ contains fewer networks than the minimum group size, we progress in the associated branch. The sizes of groups $b_{21}$ and $b_{22}$ are both smaller than $t$, so no significant subgroup is detected. Thus, we identify two significant groups in the MUTAG dataset ($a$ and $b$) with netANOVA.


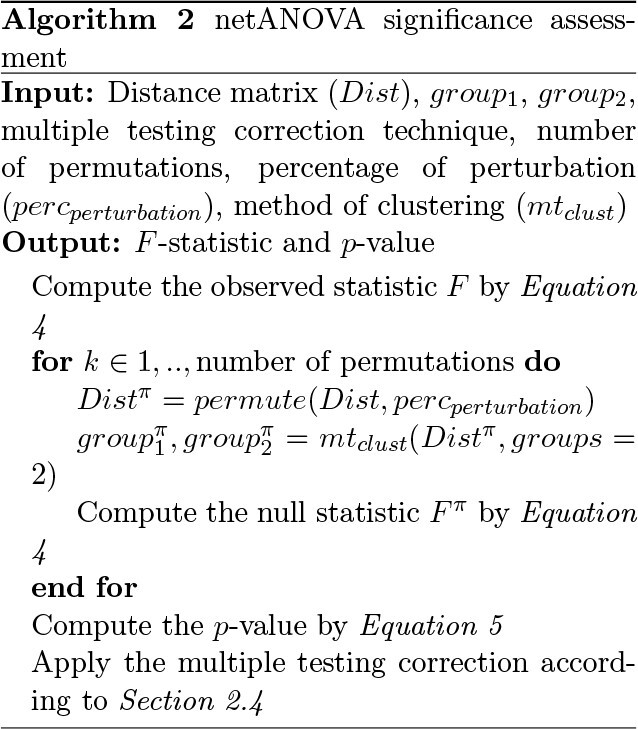


The netANOVA algorithm detected the correct number of groups (2) and the associated accuracy is 79.8% (see Table 3). Among the 10 comparative analyses, only two yielded improved performance (graPHmax and GraPHmax+NF) compared with netANOVA. Importantly, with all the methodologies except netANOVA, the correct number of groups was forced. Overall, results show that netANOVA was able to achieve competitive and in many cases superior performance while being able to determine the number of groups and assess statistical significance. 

**Table 3:** UNC scenario: clustering accuracy of unsupervised graph-based algorithms applied to the MUTAG dataset. The different unsupervised algorithms take as input the list of 188 nitroaromatic compound networks. The mutagenicity (two groups) of the compounds on Salmonella typhimurium is considered as the ground truth. These group labels are not used to derive the clusterings, but only a posteriori to obtain the accuracy by comparison between the inferred groups and the ground truth.

Method	Accuracy (%)
DGI [55]	72.34
InfoGraph [12]	77.65
GraPHmax+NF [13]	84.10
GraPHmax+EF [13]	68.08
GraPHmax-P[13]	77.12
GraPHmax-H [13]	76.59
GraPHmax [13]	85.04
graph2vec [14]	78.2
graph2vec + autoencoder [14, 56]	77.01
random walk kernel [57]	67.02
netANOVA	79.8

#### KNC scenario

The KNC application takes as input the list of 124 individual-specific brain networks. The goal is to see if we can differentiate the group of controls and the group of people with schizophrenia (two groups). We compared netANOVA performance with six approaches previously used on this dataset: graphclass [47], Elastic net [58], SVM-L1 [59], Signal-subgraph [60], DLDA and LASSO (see Table 4).

**Table 4:** KNC scenario: accuracy for different classification and clustering methods with variable selection on the COBRE dataset. The list of 124 individual-specific brain networks is used as input. The status cases (schizophrenia) and controls are considered as the ground truth. Supervised results are reported from Relión *et al*. [47]. The group labels are not used within the netANOVA clustering. They are used a priori to identify relevant graph substructures using the method developed by Relión *et al*. [47] (Section 2.6.3). The group labels are also used a posteriori to obtain the accuracy by comparison between the inferred groups and the ground truth.

	Method	Accuracy (%)
**Supervised**	graphclass [47]	92.7(2.6)
Cross-validated accuracy (average	Elastic net [58]	89.5 (1.8)
and standard errors over 10 folds)	SVM-L1 [59]	87.9 (2.2)
	Signal-subgraph [60]	86.1 (3.3)
	DLDA	84.6 (3.3)
	LASSO	80.1 (5.6)
**Unsupervised**		
	netANOVA $\rho =1$ (3,766 edges)	91.6
	netANOVA $\rho =0.8$ (4,817 edges)	100
	netANOVA $\rho =0.6$ (6,283 edges)	100
	netANOVA $\rho =0.4$ (9,606 edges)	no group detected
	netANOVA $\rho =0.2$ (33,796 edges)	no group detected
	netANOVA $\rho =0$ (34,453 edges)	no group detected

In netANOVA, we compute the pairwise distance between the 124 brain networks using the edge difference distance because nodes are labelled and weighted. The minimum group size is s10 and the default options were used. Since networks are originally fully connected, we evaluated the impact of graph sparsification using the method developed by Relión *et al*. [47] (Section 2.6.3). Hierarchical clustering is applied to the distance matrix, and the Algorithm 1 is recursively used to identify the final groups. We illustrate the procedure in Figure 3**(B)**. The first two groups are significantly different. Then, $a_1$, $a_{21}$ and $a_{221}$ are too small to be tested. Groups $a_{2221}$ and $a_{2222}$ are not significantly different. Also, $b_1$ is too small to be tested, and groups $b_{21}$ and $b_{22}$ are not significantly different. Thus, we identify two significant groups in the COBRE dataset ($a$ and $b$).

When focusing on 6000 edges or less, a minimum accuracy of 91.9% was obtained with netANOVA. However, when too many edges were considered, we could not distinguish between cases and controls. In the context of brain networks, it was already reported that feature selection is required to detect differences between groups (see Supplementary). When focusing on relevant edges, netANOVA was again among the top performers compared with supervised methods which usually lead to an inflated accuracy since the phenotype is used in the model. Hence, netANOVA was able to identify groups from a set of networks where nodes are labelled when proper edge selection was performed a priori.

## Discussion

In this article, we propose a novel workflow for statistical clustering of entire graphs, evaluate its properties (Sections 3.1 and 3.2) and validate it via biological networks use cases (Section 3.3 ). The extensive simulations show that netANOVA can reach high performance, both regarding type I error control and power, and show which option to prioritize depending on the context. The applications on real data reveal that the method achieves competitive results since netANOVA is often among the top approaches. This highlights the method’s potential in real-life situations.

Most of the components in the procedure do not require a high computing time (Supplementary). The most influential aspects are the number of networks, their density and the distance chosen to compare the graphs. Distances for graphs with no node correspondence often require a longer processing time. Also, the computations of the first permutation-based significance assessments are the most intense due to the number of graphs compared.

### Novelty of the netANOVA strategy

The workflow differs from generic non-parametric multivariate ANOVA [25] and standard clustering methods in several respects. NetANOVA is a comprehensive graph-specific clustering workflow developed on strong statistics. It takes as input a set of networks, derives potential groups, determines the optimal number of groups without the need to set externally the number and assesses statistical significance while being completely unsupervised. Although this can be a great advantage for a user, it makes our workflow difficult to compare with baselines. Indeed, common methods often perform only one part of the analysis and there is a lack and a need for such complete approaches. For instance, a common strategy in the absence of graph labels and graph comparative analysis is to generate graph embedding, such as Graph2Vec [14], and AWE [61]. These are fed into downstream models, such as a $k$-means clustering. However, deriving the optimal number of clusters is often decoupled from the mainstream analysis [13], which is not the case in our proposed workflow. GCNs [62] have also become a growing topic for supervised and unsupervised network clustering. We showed that netANOVA is able to compete and sometimes outperform GNNs approaches while bringing additional interpretability properties and being applicable to small datasets. Fraiman *et al*. [63] outline another strategy. These authors examine network differences between groups with an ANOVA test explicitly developed for networks. They test whether the mean networks for predetermined groups are the same versus the alternative that at least one group has a deviating average network. Significance is derived by randomly distributing observations across groups in which no subgroup differences are to be expected. In contrast, we do not use the notion of an average network. The reason is that such a notion is not always meaningful. Also, our proposed workflow does not assume knowledge about group formation but identifies relevant partitions on the fly. The permutation procedure is also customized to handle the hierarchical structure of the workflow. In addition, the approach includes components to control for type I error, which improves the confidence in the detected groups.

### Significance assessment

Several choices were made in the significance assessment procedure. The permutation-based significance assessment cannot be performed as in classical non-parametric distance-wise ANOVA [25] because the clusters are derived via hierarchical clustering. Even if there are no actual groups, the clustering will create it by grouping the most similar networks, decreasing the within-group variance and increasing the across-group variance. Thus, a permutation of the graph labels to compute a *P*-value will bias towards false positives. Since the significance assessment is conditional on the two groups because of the hierarchical clustering, the same data must not be used to perform clustering and assess significant differences between groups. Multiple suggestions have been suggested to tackle this issue. In Gao *et al*. [43], the authors propose a selective inference approach to test for a difference in means between two clusters. Kimes *et al*. [64] developed a Monte Carlo based approach for statistical testing significance in hierarchical clustering. Suzuki and Shimodaira [65] developed the R package pvclust where the hypothesis tests are based on bootstrapping procedures. Our approach also relies on randomization of the observed data, using permutations of the distances between the investigated graphs instead of the graph label. We re-apply the hierarchical clustering on these permuted sets to identify two groups and compare the obtained labels with the observed ones. Since the permutation of the distance has the additional impact that it no longer satisfies the triangular inequality, the linkage method in the hierarchical clustering is restricted.

### Userfriendliness of netANOVA

There are multiple options in the workflow, such as the distance, the multiple testing correction method, the hierarchical linkage criteria, the minimum size of a group to be tested, the significance threshold, the number of permutations and the percentage of distances permuted in the distance matrix. It can therefore adapt to multiple scenarios and network types. Practical considerations on the minimum group size are presented in Supplementary. The customizable properties of netANOVA make it relevant to a larger range of users. For example, even though netANOVA has been developed for network analyses, it is generic in that it can accommodate any type of object. The only prerequisite is that a meaningful pairwise distances matrix can be computed.

### Future enhancements

Our netANOVA workflow in the context of high-density networks can be improved. For now, edge selections may be required to select the most informative subnetworks and must be performed a priori. In our KNC application, the edge selection in COBRE networks is supervised and applied to the same dataset as the clustering (Section 2.6.3). Even if the clustering is then performed unsupervised, this could lead to overoptimistic performance estimates. This KNC application shows the importance of focusing on relevant interactions to improve interpretability and accuracy. Thresholding is typically adopted to cancel a percentage of the weakest connection, to turn fully connected and weighted brain networks into a useful sparse network. De Vico Fallani *et al*. [66] indicate that the way to fix this threshold is still an open issue, and they introduce a criterion, the efficiency cost optimization (ECO), to select a threshold based on the optimization of the trade-off between the efficiency of a network and its wiring cost. ‘Informative’ parts can be also extracted in non-supervised ways [67] for instance by looking for areas in the networks that exhibit a lot of variation between individuals, assuming that the more variation we have in ‘the input’, the more we will be able to explain with it. On the other hand, for weighted networks, even when we have a selection of nodes under consideration, the network will still be dense. Hence, some approaches based on multiple thresholds, such as filtration curves can be considered to capture a balance between hard thresholding and fully connected networks. The different thresholds reveal different structures in the graphs, and how these structures change from one threshold to another may be quite different from one network to another.

**Key Points**

- The identification of homogeneous groups of networks is a common problem in system medicine. Often, the group labels are unknown, and there is no knowledge about the partitioning or the number of classes. Also, there is a need to know if the groups are significantly statistically different or not to enhance the belief in the discovered groups. We addressed these hurdles by developing an unsupervised approach based on reliable statistics that considers graphs’ specificities and derives groups of similar networks.
- Personalized screening before therapy enables improving diagnostic precision and treatment results. In network medicine, there is a trend to describe patients via individual-level biological networks, where edges are individual-specific. The tool developed in this paper paves the way towards exploiting individual networks to identify relevant disease subtypes and enhance stratified medicine.
- The method is flexible and user-friendly, making it relevant to a larger range of users. There are multiple options in the workflow, such as the distance between networks, the multiple testing correction method, the hierarchical linkage criteria, the minimum size of a group to be tested, the significance threshold or the number of permutations. It can therefore adapt to multiple scenarios and network types. In addition, even though netANOVA has been developed for network analyses, it can accommodate any type of object. The only prerequisite is that a meaningful pairwise distances matrix can be computed.

## Supplementary Material

netANOVA_supplementary_revised_bbad029

supplementary_bbad029

## Data Availability

The code necessary to reproduce this article’s results and analyses is available on GitHub at https://github.com/DianeDuroux/netANOVA. The MUTAG dataset is available at https://networkrepository.com/Mutag.php [68]. The COBRE data was obtained from http://fcon_1000.projects.nitrc.org/indi/retro/cobre.html. It is available at https://rdrr.io/github/jesusdaniel/graphclass/man/COBRE.data.html [47, 49].

## Author Biographies

**Diane Duroux** is a PhD student at the University of Liège, Belgium. Her research mainly focuses on network medicine. She is interested in detecting, understanding and reducing heterogeneity between networks to enhance stratified medicine.

**Kristel Van Steen** is a Professor at the University of Liège (Belgium). She also holds a part-time professorship position in Systems Medicine at the KU Leuven Department of Human Genetics. She has built up a recognized expertise in developing and applying methods to detect gene x gene and gene x environment interactions and in unifying biological and statistical evidence in genetic epidemiology.

## References

[1] Lee B , Zhang S, Poleksic A, et al. Heterogeneous multi-layered network model for omics data integration and analysis. Front Genet 2020; 10:1381. 32063919 10.3389/fgene.2019.01381 PMC6997577

[2] Muzio G , O’Bray L, Borgwardt K. Biological network analysis with deep learning. Brief Bioinform 2021; 22(2): 1515–30. 33169146 10.1093/bib/bbaa257 PMC7986589

[3] Matsubara T , Ochiai T, Hayashida M, et al. Convolutional neural network approach to lung cancer classification integrating protein interaction network and gene expression profiles. J Bioinform Comput Biol 2019; 17(03): 1940007. 31288636 10.1142/S0219720019400079

[4] Rhee, S., Seo, S., and Kim, S. (2017) *arXiv preprint arXiv:1711.05859*.

[5] Gregorich M , Melograna F, Sunqvist M, et al. Individual-specific networks for prediction modelling – a scoping review of methods. BMC Med Res Methodol 2022; 22(1): 1–17. 35249534 10.1186/s12874-022-01544-6 PMC8898441

[6] Camacho DM , Collins KM, Powers RK, et al. Next-generation machine learning for biological networks. Cell 2018; 173(7): 1581–92. 29887378 10.1016/j.cell.2018.05.015

[7] Supekar K , Menon V, Rubin D, et al. Network analysis of intrinsic functional brain connectivity in Alzheimer’s disease. PLoS Comput Biol 2008; 4(6): e1000100. 18584043 10.1371/journal.pcbi.1000100 PMC2435273

[8] Liu Y , Liang M, Zhou Y, et al. Disrupted small-world networks in schizophrenia. Brain 2008; 131(4): 945–61. 18299296 10.1093/brain/awn018

[9] Tantardini M , Ieva F, Tajoli L, et al. Comparing methods for comparing networks. Sci Rep 2019; 9(1): 1–19. 31772246 10.1038/s41598-019-53708-y PMC6879644

[10] Borgwardt, K., Ghisu, E., Llinares-López, F., O’Bray, L., and Rieck, B. (2020) *arXiv preprint arXiv:2011.03854*.

[11] Zhou J , Cui G, Hu S, et al. Graph neural networks: a review of methods and applications. AI Open 2020; 1:57–81.

[12] Sun, F.-Y., Hoffmann, J., Verma, V., and Tang, J. (2019) *arXiv preprint arXiv:1908.01000*.

[13] Bandyopadhyay, S., Aggarwal, M., and Murty, M. N. (2020) *arXiv preprint arXiv:2006.04696*.

[14] Narayanan, A., Chandramohan, M., Venkatesan, R., Chen, L., Liu, Y., and Jaiswal, S. (2017) *arXiv preprint arXiv:1707.05005*.

[15] Defferrard M , Bresson X, Vandergheynst P. Advances in neural information processing systems 2016;29.

[16] Kipf, T. N. and Welling, M. (2016) *arXiv preprint arXiv:1609.02907*.

[17] Zampieri G , Vijayakumar S, Yaneske E, et al. Machine and deep learning meet genome-scale metabolic modeling. PLoS Comput Biol 2019; 15(7):e1007084. 10.1371/journal.pcbi.1007084 PMC6622478 31295267

[18] Miotto R , Wang F, Wang S, et al. Deep learning for healthcare: review, opportunities and challenges. Brief Bioinform 2018; 19(6): 1236–46. 28481991 10.1093/bib/bbx044 PMC6455466

[19] Ribeiro MT , Singh S, Guestrin C. Proceedings of the 22nd ACM SIGKDD international conference on knowledge discovery and data mining, 2016, 1135–44.

[20] Malin BA , Emam KE, O’Keefe CM. Biomedical data privacy: problems, perspectives, and recent advances, 2013. 10.1136/amiajnl-2012-001509 PMC3555341 23221359

[21] Niepert M , Ahmed M, Kutzkov K. International conference on machine learning PMLR, 2016, 2014–23.

[22] Yang K , Swanson K, Jin W, et al. Analyzing learned molecular representations for property prediction. J Chem Inf Model 2019; 59(8): 3370–88. 31361484 10.1021/acs.jcim.9b00237 PMC6727618

[23] Nouranizadeh, A., Matinkia, M., Rahmati, M., and Safabakhsh, R. (2021) *arXiv preprint arXiv:2107.01410*.

[24] Wu, J., Li, S., Li, J., Pan, Y., and Xu, K. (2022) *arXiv preprint arXiv:2206.02404*.

[25] Anderson MJ . A new method for non-parametric multivariate analysis of variance. Austral Ecol 2001; 26(1): 32–46.

[26] Ontañón S . An overview of distance and similarity functions for structured data. Artif Intell Rev 2020; 53(7): 5309–51.

[27] Phillips, J. M. and Venkatasubramanian, S. (2011) *arXiv preprint arXiv:1103.1625*.

[28] Bai L , Hancock ER, Torsello A, et al. International Workshop on Graph-Based Representations in Pattern Recognition Springer, 2013, 121–31.

[29] Kondor R , Pan H. Advances in neural information processing systems 2016;29. PMC5361064 28344429

[30] Nikolentzos G , Meladianos P, Limnios S, et al. In IJCAI. 2018;2595–601.

[31] Shervashidze N , Vishwanathan S, Petri T, et al. Artificial intelligence and statistics PMLR, 2009, 488–95.

[32] Yanardag P , Vishwanathan S. Proceedings of the 21th ACM SIGKDD international conference on knowledge discovery and data mining, 2015, 1365–74.

[33] Shervashidze N , Borgwardt K. Advances in neural information processing systems 2009;22.

[34] Hammond DK , Gur Y, Johnson CR. In 2013 IEEE Global Conference on Signal and Information Processing IEEE, 2013, 419–22.

[35] Sugiyama M , Borgwardt K. Advances in neural information processing systems 2015;28. PMC5467221 28615917

[36] Koutra D , Vogelstein JT, Faloutsos C. Proceedings of the 2013 SIAM International Conference on Data Mining SIAM, 2013, 162–70.

[37] Yip AM , Horvath S. In BIOCOMP. 2006;451–7.

[38] Ferwerda J , Hainmueller J, Hazlett CJ. Kernel-based regularized least squares inR(KRLS) andStata(krls). J Stat Softw 2017; 79(3): 1–26.30220889

[39] Hamming RW . Error detecting and error correcting codes. Bell Syst Tech J 1950; 29(2): 147–60.

[40] Borgwardt KM , Kriegel H-P. Fifth IEEE international conference on data mining (ICDM’05) IEEE, 2005, 8.

[41] Meinshausen N . Hierarchical testing of variable importance. Biometrika 2008; 95(2): 265–78.

[42] Girden ER . ANOVA: repeated measures, number 84sage. 1992.

[43] Gao, L. L., Bien, J., and Witten, D. (2020) *arXiv preprint arXiv:2012.02936*.

[44] Ackermann MR , Blömer J, Sohler C. Clustering for metric and nonmetric distance measures. ACM Trans Algorithms 2010; 6(4): 1–26.

[45] Csardi G , Nepusz T. Int J Complex Syst 2006;1695.

[46] Debnath AK , Lopez RL, Debnath G, et al. Structure-activity relationship of mutagenic aromatic and heteroaromatic nitro compounds. Correlation with molecular orbital energies and hydrophobicity. J Med Chem 1991; 34(2): 786–97. 1995902 10.1021/jm00106a046

[47] Relión JDA , Kessler D, Levina E, et al. Ann Appl Stat 2019; 13(3): 1648. 33408802 10.1214/19-AOAS1252 PMC7785130

[48] Meng L , Xiang J. Brain network analysis and classification based on convolutional neural network. Front Comput Neurosci 2018; 12:95. 30618690 10.3389/fncom.2018.00095 PMC6295646

[49] Aine C , Bockholt HJ, Bustillo JR, et al. Multimodal neuroimaging in schizophrenia: description and dissemination. Neuroinformatics 2017; 15(4): 343–64. 28812221 10.1007/s12021-017-9338-9 PMC5671541

[50] Power JD , Cohen AL, Nelson SM, et al. Functional network Organization of the Human Brain. Neuron 2011; 72(4): 665–78. 22099467 10.1016/j.neuron.2011.09.006 PMC3222858

[51] Wills P , Meyer FG. Metrics for graph comparison: a practitioner’s guide. PloS one 2020; 15(2): e0228728. 32050004 10.1371/journal.pone.0228728 PMC7015405

[52] Redcay E , Moran JM, Mavros PL, et al. Intrinsic functional network organization in high-functioning adolescents with autism spectrum disorder. Front Hum Neurosci 2013; 7:573. 24062673 10.3389/fnhum.2013.00573 PMC3777537

[53] Hull JV , Dokovna LB, Jacokes ZJ, et al. Resting-state functional connectivity in autism Spectrum disorders: a review. Front Psych 2017; 7:205. 10.3389/fpsyt.2016.00205 PMC5209637 28101064

[54] Gupta S , Gupta A. Dealing with noise problem in machine learning data-sets: a systematic review. Procedia Comput Sci 2019; 161:466–74.

[55] Veličković P , Fedus W, Hamilton WL, et al. arXiv preprint arXiv:1809.10341. 2018.

[56] Rumelhart DE , Hinton GE, Williams RJ. Learning internal representations by error propagation Technical report California Univ San Diego La Jolla Inst for Cognitive Science, 1985.

[57] Gärtner T , Flach P, Wrobel S. On graph kernels: Hardness results and efficient alternatives In Learning theory and kernel machines. Springer, 2003, 129–43.

[58] Friedman J , Hastie T, Tibshirani R. Regularization paths for generalized linear models via coordinate descent. J Stat Softw 2010; 33(1): 1–22. 20808728 PMC2929880

[59] Zhu J , Rosset S, Tibshirani R, et al. Advances in neural information processing systems Citeseer, 2003, p. None.

[60] Vogelstein JT , Roncal WG, Vogelstein RJ, et al. Graph classification using signal-subgraphs: applications in statistical Connectomics. IEEE Trans Pattern Anal Mach Intell 2013; 35(7): 1539–51. 23681985 10.1109/TPAMI.2012.235

[61] Ivanov S , Burnaev E. International conference on machine learning PMLR, 2018, 2186–95.

[62] Bai, Y., Ding, H., Qiao, Y., Marinovic, A., Gu, K., Chen, T., Sun, Y., and Wang, W. (2019) *arXiv preprint arXiv:1904.01098*.

[63] Fraiman D , Fraiman R. An ANOVA approach for statistical comparisons of brain networks. Sci Rep 2018; 8(1): 1–14. 29549369 10.1038/s41598-018-23152-5 PMC5856783

[64] Kimes PK , Liu Y, Neil Hayes D, et al. Statistical significance for hierarchical clustering. Biometrics 2017; 73(3): 811–21. 28099990 10.1111/biom.12647 PMC5708128

[65] Suzuki R , Shimodaira H. Pvclust: an R package for assessing the uncertainty in hierarchical clustering. Bioinformatics 2006; 22(12): 1540–2. 16595560 10.1093/bioinformatics/btl117

[66] De Vico Fallani F , Latora V, Chavez M. A topological criterion for filtering information in complex brain networks. PLoS Comput Biol 2017; 13(1):e1005305. 10.1371/journal.pcbi.1005305 PMC5268647 28076353

[67] Duroux, D., Climente-Gonzáles, H., Azencott, C.-A., and Van Steen, K. (2020) *bioRxiv*. 10.1093/gigascience/giab093 PMC8848319 35134928

[68] Rossi, R. A. and Ahmed, N. K. (2015) In AAAI

