# Supplementary material for: netANOVA: novel graph clustering technique with significance assessment via hierarchical ANOVA
Source: Brief Bioinform. 2023 Feb 4;24(2):bbad029. doi: 10.1093/bib/bbad029 (PMC10025436; doi:10.1093/bib/bbad029)
Supplement: netANOVA_supplementary_revised_bbad029 [file netanova_supplementary_revised_bbad029.pdf]

NetANOVA - Supplementary

1 Details on distances included in the workflow

Our workflow proposes multiple distances and similarity measures with different properties to allow the user to choose the one relevant to its specific use case. We included the simple edge difference distance [1] that inputs two adjacency matrices and takes the Frobenius norm of their differences as a baseline because this method is computationally fast and easy to interpret. In the same vein, we propose the Gaussian kernel [2] that is applied to the vectorised edge weights. It is defined as $k_{gaussian}(x_i, x_j) = exp(\frac{-||x_i - x_j||^2}{\sigma^2})$ where $||x_i - x_j||$ is the Euclidean distance and σ^2 is the bandwidth of the kernel. Both methods can be applied to weighted and KNC graphs. The hamming distance [3] that counts the discrepancy between two networks for each edge can also be chosen and used on binary networks. Furthermore, the user can select the shortest path kernel [4], which defines the similarity between two graphs in terms of the similarities of their shortest paths. It is the only measure proposed that is based on substructures. It is computationally intensive when the number of nodes increases. Then, we include the k-step random walk kernel [5]. It measures graph similarity by counting matching walks in two graphs. Longer walks of length k are down-weighted by a factor of $\lambda^k (\lambda < 1)$ to ensure convergence of the corresponding geometric series. It is the only method that is based on walks. Both shortest-path kernel and k-step random walk kernel can be applied on directed networks. For a specific situation where a user considers networks having the same set of fully connected nodes, we developed a customised version of the k-step random walk kernel. In that context, only node and edge attributes change from one network to another, and in fact, the graphs are represented in a feature space that is described as a graph, but it is always the same network, so one is comparing different labelling of the same graph. Hence, the random walk kernel is customised with the constraint that the node ids have to be identical. k_{RW} is defined as follow: $k_{RW}(A_i, A_j) = \sum_{k=0}^{n} \mu(k) q^T W^k p$, with W the Hadamard product (instead of Kronecker product) of A_i and A_j adjacency matrices of individuals i and j, p and q the initial and stopping probability distributions, and μ a chosen non-negative coefficient. The probabilities p and q can be vectors of 1s to compare individuals through their individual edge weights only; they can also take the node values to compare individuals through their individual edge weights and individual node weights. Another distance that a user can apply is DeltaCon [6]. The first step of the method is to compute the pairwise node affinities via Fast Belief Propagation (FABP) in the two graphs and measure the differences in the corresponding node affinity scores as their similarity score using the

root euclidean distance. DeltaCon computes graph similarity with known node correspondence, and this method is especially useful in detecting changes in the connectivity of graphs. Also, the Graph Diffusion Distances [1] is accommodated. It quantifies the difference between two graphs of the same size and is based on measuring the average similarity of heat diffusion on each graph. It takes an idea from the heat diffusion process on graphs via graph Laplacian exponential kernel matrices and can be applied to the weighted network. An advantage of Graph Diffusion Distances is that it is a metric in the strict mathematical sense. Finally, the option GTOM [7] can be selected. This measure is constructed by counting the number of m-step neighbours that a pair of nodes share and then normalised suitably. It computes a dissimilarity measure based on the notion of topological overlap. Notably, it is independent of the number of paths and the number of geodesic paths connecting a node and its m-step neighbour. Thus, the range of proposed distances allows the user to select the most relevant one depending on the specificity of its input graphs and the graph properties that he wants to capture and that can be context-specific.

2 Comparison of distance and similarity measures

We propose a large range of distances between networks in our workflow but plenty of other measures for network comparison have been developed and were reviewed in depth elsewhere. For instance, in Tantardini et al. [8], the authors evaluate the performance of such methods, carrying out clusterings. They highlight different behaviours and performances between KNC and UNC methods. When networks of the same size and density are considered, most procedures can reasonably discriminate between different structures in the undirected and directed case, often achieving a perfect prediction. However, the results change considerably when considering different sizes and densities of networks. In both the undirected and the directed cases, the graphlet-based measures GCD-11 and DGCD-129 demonstrate superior performance to the other methods in discriminating between different network topologies and outperforming all the other methods investigated. In Wills et al. [9], the authors observe that the adjacency spectral distance exhibits good performance for the comparison graphs of different sizes and the comparison of graphs without known vertex correspondence. Shimada et al. [10] also highlight that graph distance based on the comparison of their Laplacian matrices is interesting because the Laplacian matrix contains essential information about the structural and dynamical properties of networks. In addition, when examining the global structure, Wills et al. [9] find that the adjacency spectral distance and DeltaCon distance both provide good performance. These authors recommend using a spectral distance computed from either the combinatorial graph Laplacian or the adjacency matrix to distinguish graphs via their mesoscale connectivity structures. However, the adjacency spectral distance is not the most appropriate choice in any situation. Wills et al. [9] studied the problem of detecting change points in a dynamic graph. To detect change points, the distance between consecutive time steps is calculated. In that context, the two compared networks share many more edges than in the usual two-sample test. The matrix distances such as the resistance perturbation

distance, or DeltaCon, give very high performance and perform better when detecting changes in the network dynamics variables. In the meantime, the spectral distances, yield deficient performance. Therefore, it is crucial to know whether local topological features are of interest in the graph comparison. If local structures are not informative, selecting distances focusing on such structures can harm the accuracy. On the other hand, important information can be lost when local structure is not considered. Hence, the choice of the distance is domain-specific, will depend on the nature of the networks and will vary with the information one is interested in. For instance, the user may initially focus on large-scale network structures (e.g. community structure or the hubs), or small scale features (e.g. local connectivity or triangle graphlets). We recommend to test multiple distance measures and combine the analysis of graph structures to derive a consensus [11].

3 Clustering

We selected the hierarchical clustering to group networks based on distances, but a multitude of algorithms exist that enable clustering. Here, we discuss the main categories of clustering methods: hierarchical, partitional, grid, density-based, and model-based. Hierarchical clustering forms groups by iteratively dividing or aggregating the objects in a top-down or bottom-up manner. Partitional clustering optimizes some criterion functions such as the Euclidean distance between the object with each of the available clusters. It assigns the object to the closest cluster (k-means [12, 13], fuzzy k-means [14]). Grid clusterings [15] partition the objects into a finite number of cells to create a grid structure and derive groups from these cells. In density-based methods, clusters are separated from other groups other by contiguous regions of low point density (DBSCAN [16, 17], Optics [18]). In mixture density-based methods, objects are assumed to be generated from probability distributions and can be derived from several categories of density functions, or from the same families but with different parameters. Spectral clustering [19] uses information from the eigenvalues of special matrices built from the graph or the data set. Probabilistic clustering (Bayes framework [20]) uses Gibbs posteriors to improve the quantification of uncertainty in the estimated clusters, face computational problems, and large sensitivity to the choice of kernel.

The different methods and their associated aims and assumptions can confuse the user. Many reviews [21, 22, 23, 24, 25] compare approaches. Overall, they underline that no clustering algorithm universally outperforms the others in all contexts. Whereas optimality is often defined in terms of excellent cluster separation and within-cluster homogeneity for an increasing number of applications in biomedicine, the best clustering algorithms will be able to deal with a lot of objects and high-dimensional features. In the meantime, it will be scalable, in terms of storage requirements and running. It should identify irregular shapes of classes and handle outliers and noise. The most relevant methods will also decrease their reliance on parameters that are set by the users. Optimally, the clustering would be able to deal with new data without recomputing the classes from scratch. It will not be dependent on the order of the input patterns. It will handle multiple data types, such as quantitative and qualitative inputs. Also, it should give a reasonable estimation of the final

Table 1: Parameter choices in simulations and real life applications. In bold, default parameters of the R function.

Parameter	Type I error	Power	Real life KNC	Real life UNC
# Networks	50-100	20-500	188	124
Density	0.05-0.1	0.05-0.1	0.08 to 0.22	0.11 to 1
Network type	random	random, scale-free, cluster	NA	NA
#Nodes	100-500	100	10 to 28	263
Edge type	Weigted, unweighted	Weighted, unweighted	Unweighted	Weighted
# Groups	0	2-10	2	2
Distance	All	All	Random Walk kernel	Edge difference
Multiple testing	NA	**tree**-Meinshausen	**tree**	**tree**
HC criteria	**Complete**-Average	**Complete**-Average	**Complete**	**Complete**
Minimum group size	5-10	5	40	10
Significance threshold	**0.05**	**0.05**	**0.05**	**0.05**
# permutations	**99**	**99**	**99**	**99**
% Distances permuted	10-**20**-50	**20**	**20**	**20**

number of clusters without prior knowledge. Finally, it will provide relevant data visualization.

In netANOVA, we chose hierarchical clustering for the following reasons. This algorithm can detect arbitrary cluster shapes rather than being restricted to common shapes. It is insensitive to the order of input patterns and doesn't rely on a large number of parameters. In addition, whereas we only input continuous values (distances) in our examples, it accepts different data types which makes it easily extendable to different contexts. Strikingly, it provides results as an informative tree structure that is readily interpretable. This visualization helps in understanding and identifying the number of clusters.

4 Comparison of UNC and KNC inputs and outcomes

The two real-life data application settings are very different. In the MUTAG dataset, networks are smaller with about 28 nodes, and categorical edges are recorded with no noise. In this contewt, graph clustering will look for discriminative patterns, such as communities or paths. This is computationally feasible only on small graphs. The application of netANOVA on this set of networks with exchangeable nodes gives rise to competitive properties. With graphs without exchangeable nodes, networks are larger (263 nodes). The graphs are initially fully connected, the edges are weighted and may contain noise since they are statistically derived. We found that there is a need to focus on relevant communities or edges to achieve high clustering performance. Wills et al. [9] come to the same conclusion. They compare sets of connectomes in two types of analysis: weighted

Table 2: Properties of networks and parameters used to derive results presented in Figure 2. The baseline scenario has an original network with a random structure, 100 nodes, a density of 0.05 and binary edges. It contains 10 groups and 10 networks per group obtained via degree preserving rewiring 40% of the edges. Hence, in the column "Type of edge pertubation", the value "DPR" stands for "Degree Preserving Rewiring". The hierarchical clustering is performed with complete-linkage clustering and the multiple testing correction is based on the depth of the dendrogram. In the other scenarios, we altered one parameter at a time.

	# individual networks per group	Density of the original network	Type of the original network	Edge type	# Groups	Multiple testing	hierachical clustering criteria	Type of edge perturbation	Percentage of edge perturbed
Baseline	10	0.05	random	binary	10	tree	Complete	DPR	40
Density	10	0.1	random	binary	10	tree	Complete	DPR	40
Cluster	10	NA	cluster	binary	10	tree	Complete	DPR	40
Scale-free	10	NA	scale-free	binary	10	tree	Complete	DPR	40
Weighted	10	0.05	random	weighted	10	tree	Complete	DPR	40
Perturbation	10	0.05	random	binary	10	tree	Complete	DPR	60
Add	10	0.05	random	binary	10	tree	Complete	Addition	40
Switch	10	0.05	random	binary	10	tree	Complete	Random switch	40
Remove	10	0.05	random	binary	10	tree	Complete	Removal	40
Linkage	10	0.05	random	binary	10	tree	Average	DPR	40
50 networks	50	0.05	random	binary	10	tree	Complete	DPR	40
2 groups	10	0.05	random	binary	2	tree	Complete	DPR	40
MT	10	0.05	random	binary	10	Meinshausen	Complete	DPR	40

connectomes and unweighted connectomes. They vary the density of edges using two thresholds to set functional connectivity. They observe that the variability in the control population is greater than the contrast between the patients with Autism Spectrum disorders and controls populations, and hence no tested distance separates the two ensembles of connectomes. They highlight that the first reason is that only a subset of edges represent the structural differences between the two graph groups so that the dissimilarities cannot be identified if one uses all the edges. The second reason is that in the studied connectomes, the local changes in connectivity are of the same order of magnitude as the random local variations. Signal-to-noise is, in fact, a recurrent issue in analysing real-life graph data, and particularly in the context of connectivity networks of human brain activity [26]. In the presence of noise, many metrics cannot detect subtle structural differences. We note that others have reported similar findings [27, 28].

5 Brain network analyzes

In brain networks studies, two main approaches are used to compare the graphs. The first one is based on summary measures representing graph topology and ignoring edge weights. It reduces the network to global summary statistics, for instance the average degree, clustering coefficient,

or average path length, and use them as new variables. Previous studies [29, 30] have reported significant differences on this type of summary measures for groups of patients with brain diseases compared with controls. Nevertheless, this procedure ignores local structures and can't distinguish local dissimilarities which may decrease the performance of the clustering. The second approach is to consider all edge weights as a vector. This vector can be inputted into many existing clustering methods. These methods can perform well but it cannot account for network structure either. When feature selection is applied, it can give interpretability at the edge level, but it is often less relevant than identifying differentiating nodes or communities. Instead, we recommend to use feature selection methods derived specifically for graph. For instance, in Arroyo-Relión et al. [31], the authors developed a method that incorporates the network nature of the data via penalties to promote sparsity in the number of nodes, in addition to sparsity penalties that encourage selection of edges. We also recommend to apply KNC methods to study dissimilarities between brain networks such as edge difference distance, DeltaCon or GTOM [1, 6, 7].

6 UNC application: graph2vec and autoencoder

We applied the graph2vec [32] algorithm on the MUTAG dataset. We used the Geo2DR Python library [33] for constructing methods capable of learning distributed representations of graphs. We tested different values of epochs (50, 100, 150, and 200), batches (25, 50, 100, and 1000), and features (32, 64, 128, and 256). The best results are obtained with 150 epochs, 50 batches, and 128 features. The corresponding accuracy is 78.2.

We also applied graph2vec to convert variable-size graphs into a fixed-size representation of graphs and combined it with an autoencoder to test if it improves the performance. We used the following parameters: 150 epochs, 50 batches, and 128 features. We obtain an accuracy of 77.01.

7 Comparison of the computing time

We monitored the durations of several analyses performed on the MUTAG dataset 3. We compared them to the duration of the analysis performed with netANOVA. The k-means algorithm applied to the similarity matrix obtained with the random walk kernel is the fastest analysis, taking about 2 minutes and 30 seconds to run. NetANOVA takes about 5 minutes. The combination of graph2vec, autoencoder and k-means takes 26 minutes.

8 Practical consideration on the minimum group size

The choice of the minimum group sizes is restricted by the context and the size of the dataset. We study the evolution of the number of groups detected according to the minimum group size in the UNC and KNC applications (Fig. 1). We observe that we find the correct number of groups

Table 3: Computing time of the clustering performed on the MUTAG dataset. Analyses are conducted on a Scientific Linux release 7.2 (Nitrogen) cluster. The reported times were obtained with the Linux time command

	real	user	sys
netANOVA	5m3.434s	4m59.558s	0m0.985s
random walk kernel + kmeans	2m32.904s	2m29.778s	0m0.818s
graph2vec + kmeans	25m23.309s	12m21.711s	2m30.056s
graph2vec + autoencoder + kmeans	26m35.192s	12m29.419s	2m31.622s

in most scenarios. Reducing the minimum group size parameter increases the number of groups detected in the MUTAG application. Note that these groups are not necessarily false positives as they may highlight relevant differences different from the one considered as ground truth in the paper. With the ground truths selected (i.e. mutagenicity for MUTAG and case/control in COBRE datasets), the size of the smallest group is 63 for the MUTAG dataset and 54 for the COBRE dataset. Hence, a minimum group size higher than 63 for the MUTAG dataset and higher than 54 for the COBRE dataset would not allow identifying the exact groups. Overall, if there is absolutely no prior knowledge on the number of groups, we recommend testing multiple thresholds and evaluating the convergence and the trend of the number of groups detected.

References

[1] Hammond, D. K., Gur, Y., and Johnson, C. R. (2013) Graph diffusion distance: A difference measure for weighted graphs based on the graph Laplacian exponential kernel. In *2013 IEEE Global Conference on Signal and Information Processing* IEEE pp. 419–422.

[2] Ferwerda, J., Hainmueller, J., and Hazlett, C. J. (2017) Kernel-Based Regularized Least Squares in R (KRLS) and Stata (krls). *Journal of Statistical Software,* **79**(3), 1–26.

[3] Hamming, R. W. (1950) Error detecting and error correcting codes. *The Bell system technical journal,* **29**(2), 147–160.

[4] Borgwardt, K. M. and Kriegel, H.-P. (2005) Shortest-path kernels on graphs. In *Fifth IEEE international conference on data mining (ICDM'05)* IEEE pp. 8–pp.

[5] Sugiyama, M. and Borgwardt, K. (2015) Halting in random walk kernels. *Advances in neural information processing systems,* **28**, 1639–1647.

[6] Koutra, D., Vogelstein, J. T., and Faloutsos, C. (2013) Deltacon: A principled massive-graph similarity function. In *Proceedings of the 2013 SIAM International Conference on Data Mining* SIAM pp. 162–170.

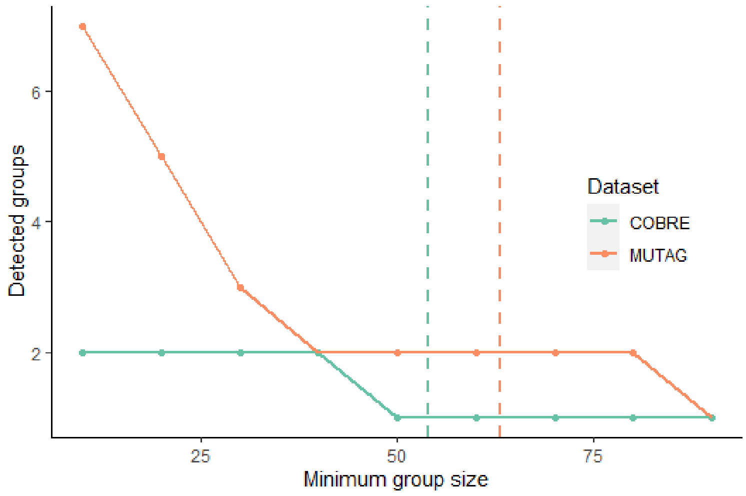

Fig. 1: Comparison of the number of groups detected with netANOVA depending on the minimum group size set for the MUTAG and the COBRE datasets. With the COBRE dataset, the edge selection was performed according to Relión et al. [31], with $\rho = 1$. The vertical dashed lines show the size of the smallest group in each dataset with the ground truth selected (i.e. mutagenicity for MUTAG and case/control in COBRE datasets). According to this ground truth, the true number of groups is 2 in each dataset.

[7] Yip, A. M. and Horvath, S. (2006) The Generalized Topological Overlap Matrix for Detecting Modules in Gene Networks.. In *BIOCOMP* pp. 451–457.

[8] Tantardini, M., Ieva, F., Tajoli, L., and Piccardi, C. (2019) Comparing methods for comparing networks. *Scientific reports,* **9**(1), 1–19.

[9] Wills, P. and Meyer, F. G. (2020) Metrics for graph comparison: a practitioner's guide. *PloS one,* **15**(2), e0228728.

[10] Shimada, Y., Hirata, Y., Ikeguchi, T., and Aihara, K. (2016) Graph distance for complex networks. *Scientific reports,* **6**(1), 1–6.

[11] Monti, S., Tamayo, P., Mesirov, J., and Golub, T. (2003) Consensus clustering: a resampling-based method for class discovery and visualization of gene expression microarray data. *Machine learning,* **52**(1), 91–118.

[12] Lloyd, S. (1982) Least squares quantization in PCM. *IEEE transactions on information theory,* **28**(2), 129–137.

[13] MacQueen, J. et al. (1967) Some methods for classification and analysis of multivariate observations. In *Proceedings of the fifth Berkeley symposium on mathematical statistics and probability* Oakland, CA, USA Vol. 1, pp. 281–297.

[14] Yang, M.-S. (1993) A survey of fuzzy clustering. *Mathematical and Computer modelling,* **18**(11), 1–16.

[15] Park, N. H. and Lee, W. S. (2004) Statistical grid-based clustering over data streams. *Acm Sigmod Record,* **33**(1), 32–37.

[16] Ester, M., Kriegel, H.-P., Sander, J., Xu, X., et al. (1996) A density-based algorithm for discovering clusters in large spatial databases with noise.. In *kdd* Vol. 96, pp. 226–231.

[17] Xie, Y. and Shekhar, S. (2019) Significant DBSCAN towards statistically robust clustering. In *Proceedings of the 16th International Symposium on Spatial and Temporal Databases* pp. 31–40.

[18] Ankerst, M., Breunig, M. M., Kriegel, H.-P., and Sander, J. (1999) OPTICS: Ordering points to identify the clustering structure. *ACM Sigmod record,* **28**(2), 49–60.

[19] Ng, A., Jordan, M., and Weiss, Y. (2001) On spectral clustering: Analysis and an algorithm. *Advances in neural information processing systems,* **14**.

[20] Rigon, T., Herring, A. H., and Dunson, D. B. (2020) A generalized Bayes framework for probabilistic clustering. *arXiv preprint arXiv:2006.05451,*.

[21] Saxena, A., Prasad, M., Gupta, A., Bharill, N., Patel, O. P., Tiwari, A., Er, M. J., Ding, W., and Lin, C.-T. (2017) A review of clustering techniques and developments. *Neurocomputing,* **267**, 664–681.

[22] Jain, A. K., Murty, M. N., and Flynn, P. J. (1999) Data clustering: a review. *ACM computing surveys (CSUR),* **31**(3), 264–323.

[23] Xu, R. and Wunsch, D. (2005) Survey of clustering algorithms. *IEEE Transactions on neural networks,* **16**(3), 645–678.

[24] Xu, R. and Wunsch, D. C. (2010) Clustering algorithms in biomedical research: a review. *IEEE reviews in biomedical engineering,* **3**, 120–154.

[25] Nagpal, A., Jatain, A., and Gaur, D. (2013) Review based on data clustering algorithms. In *2013 IEEE conference on information & communication technologies* IEEE pp. 298–303.

[26] Burgess, G. C., Kandala, S., Nolan, D., Laumann, T. O., Power, J. D., Adeyemo, B., Harms, M. P., Petersen, S. E., and Barch, D. M. (2016) Evaluation of denoising strategies to address motion-correlated artifacts in resting-state functional magnetic resonance imaging data from the human connectome project. *Brain connectivity,* **6**(9), 669–680.

[27] Redcay, E., Moran, J. M., Mavros, P. L., Tager-Flusberg, H., Gabrieli, J. D., and Whitfield-Gabrieli, S. (2013) Intrinsic functional network organization in high-functioning adolescents with autism spectrum disorder. *Frontiers in human neuroscience,* **7**, 573.

[28] Hull, J. V., Dokovna, L. B., Jacokes, Z. J., Torgerson, C. M., Irimia, A., and Van Horn, J. D. (2017) Resting-state functional connectivity in autism spectrum disorders: a review. *Frontiers in psychiatry,* **7**, 205.

[29] Supekar, K., Menon, V., Rubin, D., Musen, M., and Greicius, M. D. (2008) Network analysis of intrinsic functional brain connectivity in Alzheimer's disease. *PLoS computational biology,* **4**(6), e1000100.

[30] Liu, Y., Liang, M., Zhou, Y., He, Y., Hao, Y., Song, M., Yu, C., Liu, H., Liu, Z., and Jiang, T. (2008) Disrupted small-world networks in schizophrenia. *Brain,* **131**(4), 945–961.

[31] Relión, J. D. A., Kessler, D., Levina, E., and Taylor, S. F. (2019) Network classification with applications to brain connectomics. *The annals of applied statistics,* **13**(3), 1648.

[32] Narayanan, A., Chandramohan, M., Venkatesan, R., Chen, L., Liu, Y., and Jaiswal, S. (2017) graph2vec: Learning distributed representations of graphs. *arXiv preprint arXiv:1707.05005,*.

[33] Scherer, P. and Lio, P. Learning distributed representations of graphs with Geo2DR. (2020).
